# Highly restricted dispersal in habitat-forming seaweed may impede natural recovery of disturbed populations

**DOI:** 10.1038/s41598-021-96027-x

**Published:** 2021-08-18

**Authors:** Florentine Riquet, Christiane-Arnilda De Kuyper, Cécile Fauvelot, Laura Airoldi, Serge Planes, Simonetta Fraschetti, Vesna Mačić, Nataliya Milchakova, Luisa Mangialajo, Lorraine Bottin

**Affiliations:** 1 Institut de Recherche pour le Développement (IRD), UMR ENTROPIE, Nouméa, New Caledonia; 2 Sorbonne Université, CNRS, UMR LOV, Villefranche‑sur‑Mer, France; 3 Université Côte d’Azur, CNRS, UMR 7035 ECOSEAS, Nice, France; 4 Department of Biology, Chioggia Hydrobiological Station Umberto D’Ancona, University of Padova, Chioggia, Italy; 5 Department of Biological, Geological, and Environmental Sciences, University of Bologna, UO CoNISMa, Ravenna, Italy; 6 PSL Research University, EPHE-UPVD-CNRS, USR 3278 CRIOBE, Université de Perpignan, Perpignan, France; 7 Department of Biology, University of Naples Federico II, Naples, Italy; 8 Stazione Zoologica Anton Dohrn, Naples, Italy; 9 CoNISMa, Rome, Italy; 10 Institut za biologiju mora, Univerzitet Crne Gore, Kotor, Montenegro; 11 Laboratory of Phytoresources, Kovalevsky Institute of Biology of the Southern Seas of RAS (IBSS), Sevastopol, Russia

**Keywords:** Evolution, Evolutionary genetics, Population genetics, Ecology, Biodiversity, Conservation biology

## Abstract

*Cystoseira *sensu lato (Class Phaeophyceae, Order Fucales, Family Sargassaceae) forests play a central role in marine Mediterranean ecosystems. Over the last decades, *Cystoseira *s.l. suffered from a severe loss as a result of multiple anthropogenic stressors. In particular, *Gongolaria barbata* has faced multiple human-induced threats, and, despite its ecological importance in structuring rocky communities and hosting a large number of species, the natural recovery of *G. barbata* depleted populations is uncertain. Here, we used nine microsatellite loci specifically developed for *G. barbata* to assess the genetic diversity of this species and its genetic connectivity among fifteen sites located in the Ionian, the Adriatic and the Black Seas. In line with strong and significant heterozygosity deficiencies across loci, likely explained by Wahlund effect, high genetic structure was observed among the three seas (ENA corrected F_ST_ = 0.355, IC = [0.283, 0.440]), with an estimated dispersal distance per generation smaller than 600 m, both in the Adriatic and Black Sea. This strong genetic structure likely results from restricted gene flow driven by geographic distances and limited dispersal abilities, along with genetic drift within isolated populations. The presence of genetically disconnected populations at small spatial scales (< 10 km) has important implications for the identification of relevant conservation and management measures for *G. barbata*: each population should be considered as separated evolutionary units with dedicated conservation efforts.

## Introduction

Marine forests (i.e. seascapes dominated by habitat-forming seaweeds^1^) are among the most productive ecosystems in temperate rocky coasts, enhancing biodiversity, ecosystem functioning and habitat complexity^2,3^. However, multiple anthropogenic stressors interacting at both local and global scales increasingly threaten these iconic ecosystems^4^. As a result, a severe decline of large seaweeds abundance has been observed across temperate coastal systems over the last decades^5–8^. In particular, *Cystoseira *sensu lato (Class Phaeophyceae, Order Fucales, Family Sargassaceae), which forms the most extensive macroalgae forest in the Mediterranean Sea^9,10^, have recently suffered from irreversible loss^11^, likely underestimated as much of it may have gone unnoticed^12,13^.

Facing the dramatic decline of *Cystoseira *s.l. populations throughout the Mediterranean Sea, *Cystoseira*-formed habitats are under the protection of the Resolution 4 of the Bern Convention (Council of Europe 2010), EU Habitats Directive (The Council of the European Union 1992) and are listed in the European red list of habitats^14^. The resulting need of setting conservation actions for *Cystoseira *s.l. has recently fostered the development of ecological restoration programs: they aim to increase the effectiveness of conservation strategies of *Cystoseira *s.l., by notably including fostering natural recovery, reforesting lost areas, and/or enhancing the performance of existing human infrastructures as substrata for colonisation^15–22^*.* In addition, restoration actions also include local measures to control the drivers of canopy loss (e.g. excess of sediments and/or nutrients^5^, habitat destruction^23,24^), such as improving water quality^25^ or restoring habitat locally^12^. Such actions intend to increase the natural recovery rate of disturbed populations of *Cystoseira *s.l., expected to be very low, notably due to their life cycle comparable to a one-phase life cycle. Indeed, although *Cystoseira *s.l. display a biphasic life cycle, alternating a haploid phase with a diploid phase, the gametophytic stage, i.e. the haploid phase, is highly reduced and retained in the conceptacles, so that plants superficially regenerate only from sporophytes^26^. In addition, relatively large and heavy eggs and zygotes tend to rapidly sink just after being released^27^. As a result, *Cystoseira* likely display very limited dispersal abilities^28–30^, hampering the natural recovery of the populations.

In parallel to encouraging restoration actions, expanded research efforts regarding *Cystoseira *s.l. species distribution^31,32^ and ecology^10,30,33–35^ constantly improve scientific-based conservation plans. The diversity and phylogenetic relationships among the *Cystoseira* taxa from the eastern Atlantic and Mediterranean have also very recently been reappraised^36–38^. Nevertheless, knowledge of the genetic diversity and connectivity of natural populations further needs to be improved in order to assess the potential resilience of disturbed populations. Knowledge of the genetic variation in a study species could also help designing new restorations actions that are “future proofed” against rapidly changing conditions, such as assisted gene flow, or habitat restorations in sites according to the spatial pattern of genetic variation observed, i.e. prioritizing sites where genetic diversity would be seriously eroded.

On the one hand, genetic diversity, defined as the diversity of the basic units of hereditary information (genes) within a species, is one of the three biological diversity components, receiving increased consideration in IUCN planning and actions^14^. Beyond its significant effect on a number of ecological processes, including population recovery from disturbance and community structure^39–43^, genetic diversity is required for populations to evolve in response to ubiquitous, natural environmental change^44^. On the other hand, population connectivity, defined as the exchange of individuals among geographically separated subpopulations, plays a fundamental role in local and metapopulation dynamics, community dynamics and structure, and the resiliency of populations^45^. It ensures the genetic cohesion of a biological species over its distribution range^46^ because migration is the evolutionary force that spreads genetic diversity among populations, leading to relatively similar allele frequencies and counterbalancing the negative effect of genetic drift on genetic diversity^47^.

Severe and sudden reductions in population size are expected to result in a loss of genetic diversity and increased inbreeding^44^. In species restricted to small and isolated remnant populations, notably due to the fragmentation or destruction of their habitat, these threats can be worsened, further reducing the ability of populations to cope with environmental changes and hence increasing their risk of local extinction^48^. Nonetheless, the risk of population extinction can be reversed by migration processes^49^, making population connectivity a key feature for the recovery of the demographic structure and genetic diversity of disturbed populations. To avoid the extirpation of disturbed populations, scientific-based genetic management measures are therefore warranted^50^. As such, inferring population connectivity is a first step as the degree of connectivity among geographic areas sets the scale at which conservation strategies for marine species need to be applied^51^.

Population connectivity can be inferred from the analysis of the genetic differentiation among populations^52^. Population genetics thus offer a conceptual framework that provides increasingly appropriate statistical analysis methods to determine intra-population genetic variation, assess genetic connectivity in marine environment and infer demography history of populations^48,53^. In *Cystoseira *s.l., high levels of genetic structure indicative of low population connectivity have been observed in several species^11,13,54–58^ in accordance with their expected poor dispersal abilities.

In the Mediterranean Sea, *Gongolaria barbata* (Stackhouse) Kuntze, formerly known as *Cystoseira barbata* (Stackhouse) C. Agardh then *Treptacantha barbata* (Stackhouse)^59^, is one of the widely distributed Mediterranean *Cystoseira *s.l., thriving in shallow sheltered coasts and lagoons^36^. Local populations of *G. barbata* have recently suffered from spectacular regression in several areas of the Mediterranean Sea^6,15,60,61^, as well as in the Black Sea, where it was formerly a dominant species^62,63^. This species is severely threatened both by land-based and marine human impacts, such as the artificialization of the coastline which likely appears more important in sheltered bays than in exposed shores. The decrease in water quality, including pollution, eutrophication and increase in sediment loads, provides an additional threat whose effects are particularly important in enclosed bays with low water renewal. As a result, this species is now only found in few localities across its distribution range^64^. The combination of geographic isolation of remnant populations, recent decreases in abundance and expected restricted dispersal abilities in *G. barbata* call for an urgent exploration of its genetic status.

The only genetic work involving *G. barbata* samples from several locations showed that Black Sea mitochondrial DNA (mtDNA) sequences of the cytochrome oxidase subunit 1 (COI) gene systematically differed by one nucleotide from Mediterranean sequences^62^, indicating restricted gene flow between these two seas. In addition, very low intra-specific diversity was found among the Adriatic Sea, Menorca, Sicily and Black Sea samples. However, as the mtDNA lacks the necessary resolution to provide efficient detection of genetic structure^65^, the use of nuclear makers with higher polymorphism level is warranted. Microsatellite markers would likely unravel the population genetic diversity and structure in *G. barbata*, thanks to their codominance and high level of polymorphism properties^66^.

In the present study, we investigated the genetic diversity and structure of 15 relict populations sampled in the Ionian, the Adriatic and the Black Seas based on highly polymorphic markers specifically developed for the study species. This will inform whether the natural recovery of *G. barbata* depleted populations will strongly be compromised without enhanced restoration actions.

## Results

**Figure 1:**
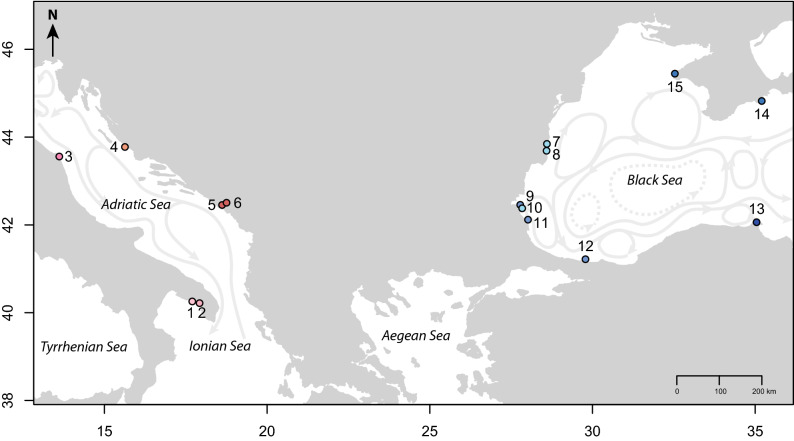
Sampling locations of *Gongolaria barbata* and the global Sea surface circulation (grey arrows). Each study site is labeled as follow: (A) in the Ionian Sea: 1—Porto Cesareo—Isola Scoglio (Italy), 2—Porto Cesareo (Italy), (B) in the Adriatic Sea: 3—Conero (Italy), 4—Kornati (Croatia), 5- and 6—Boka Kotorska (Montenegro), and (C) in the Black Sea: 7—Costinesti (Romania), 8—Kaliakra (Bulgaria), 9–11—Ropotamo Kiten (Bulgaria), 12—Sile (Turkey), 13—Sinop (Turkey), 14—Karadagand (Ukraine) and 15—Tarhankut (Ukraine). GPS coordinates are provided in Table 1. The map was created using the R package mapdata^105–107^.

A total of 503 individuals from 15 sampled populations (Fig. 1; Table 1) was amplified using the set of twelve microsatellite markers newly developed; 383 individuals successfully amplified at six markers at least, with up to two alleles per marker. Out of these 383 genotyped individuals, eight pairs of individuals (2.1%) were presumed to be clones. These were observed across several distinct sites (two pairs in site 5, in site 9 and in site 12; one pair in site 7 and in site 8). In addition, these pairs of identical individuals were labeled with sequential—or very close—numbers. As we can not rule out that the same individual was sampled twice, we decided that one unique genotype per clone was retained for further analysis. This resulted in a final dataset composed of 375 unique genotypes using twelve microsatellite markers.

**Table 1:** Genetic diversity indices of the 15 sampled sites using nine microsatellite markers.

Label	Sea	GPS coordinates		N	N_all_	A_r_	H_S_	F_IS_	g_2_
1	Ionian	40.256415	17.891478	33	5.6	3.2	0.546	0.018	0.017
2	Ionian	40.254492	17.905648	29	4.8	2.8	0.459	0.054	− 0.020
3	Adriatic	43.5575	13.612778	26	5.1	3.5	0.610	0.200***	0.015
4	Adriatic	43.7752778	15.63083333	15	3.8	2.9	0.524	0.269**	0.151**
5	Adriatic	42.453333	18.616111	35	3.9	2.4	0.429	0.228***	− 0.055
6	Adriatic	42.504733	18.753153	31	2.9	2.0	0.300	0.101	0.024
*Mediterranean subsamples*				*169*	*9.6*	*8.2*	*0.632*	0.348***	*0.056*
7	Black Sea	43.84458889	28.60035833	33	3.7	2.2	0.339	0.076***	0.080
8	Black Sea	43.412043	28.350023	31	5.0	2.7	0.419	0.048	− 0.031
9	Black Sea	42.375633	27.717717	19	4.2	2.9	0.506	0.049	− 0.028
10	Black Sea	42.331039	27.75545	11	2.9	2.4	0.424	0.048	0.093
11	Black Sea	42.186983	27.845117	2	2.1	–	0.611	0.364	–
12	Black Sea	41.18025	29.61113333	14	4.1	3.0	0.487	0.094	0.052
13	Black Sea	42.06083611	35.04415833	35	4.0	2.4	0.449	0.103***	− 0.092
14	Black Sea	44.5775	35.20333333	31	3.2	1.9	0.272	− 0.044	0.007
15	Black Sea	45.44527778	32.53277778	30	3.2	2.1	0.347	0.028	− 0.155
*Black Sea subsamples*				*206*	*9.1*	*6.2*	*0.460*	0.193***	− *0.010*
**All subsamples**				**375**	**12.9**	**3.9**	**0.441**	0.109***	**0.026**

Results for all Mediterranean subsamples on the one hand, and Black Seas subsamples on the other hand, are in italics. Results for all subsamples are in bold.

N: number of biallelic individuals successfully genotyped, N_all_: mean number of alleles per locus, A_r_: allelic richness based on minimum sample size of 5 diploid individuals, removing sample 11, H_S_: gene diversity, F_IS_: fixation index, g_2_: estimate of the identity disequilibrium.

*P-values from U test for heterozygote deficiency and for g_2_ = 0 < 0.05, **p-values < 0.01 and ***p-values < 0.001.

### Quality control of the newly developed markers

The quality and variability of the microsatellite markers were tested for population genetic studies of *G. barbata* based on a sampling comprising 15 sites in three Seas, the Ionian, the Adriatic and the Black Seas. All loci were found to be polymorphic (5–39 alleles, Table 2), with H_s_ estimates varying from 0.242 (Cysbar2) to 0.736 (Cysbar5). F_IS_ values ranged from − 0.710 (Cysbar6) to 0.501 (Cysbar3), with significant deviations from Hardy–Weinberg Equilibrium observed for seven loci (Cysbar3–Cysbar6, Cysbar9–Cysbar11). Missing data were present in all subsamples and in ten out of the 12 microsatellite markers (Supporting Information SI1), likely attributed to the presence of null alleles. Of the twelve microsatellite markers tested, MicroChecker suggested the presence of null alleles in one to five subsamples (out of 15) for seven loci (Cysbar1 to Cysbar5, Cysbar10 and 11). However, using unilateral exact binomial tests, heterozygosity deficiencies were likely explained by the presence of null alleles in only Cysbar3 (*p*-value = 0.133). MicroChecker did not detect any stuttering or short allele dominance (SAD), however, the regression approaches detected significant SAD in Cysbar5 (ρ_FIS_ = − 0.530, *p*-value = 0.0002; ρ_FST_ = − 0.591, *p*-value = 0.00003). Stuttering was not detected following De Meeûs et al*.*^67^ approach.

**Table 2:** Characterization of twelve microsatellite markers developed in *Gongolaria barbata* (15 sites sampled, N = 375 unique genotypes).

Locus (multiplex)	GenBank accession no	Primer sequences 5′–3′ and ABI fluorescent dyes	Repeat type	Size	N_a_	H_S_	F_IS_
Cysbar1 (1)	KT344783	F:6FAM-ATACAGTAATGAATGGTACAGCG	(TG)_12_	169–204	15	0.508	0.042
		R:ACATTCTGCTACGGGGACAG					
Cysbar2 (1)	KT344784	F:6FAM-AGATCCTCTGAAAGGCGGTC	(TG)_13_	87–130	10	0.242	0.099
		R:ACTCTGCGACGTTCCTGTAG					
**Cysbar3 (1)**	**KT344785**	**F:ROX-GCAGGAGTCCGGTGCTAC**	**(AC)**_**21**_	**141–212**	**18**	**0.415**	0.501***
		**R:CTATCACGGGTTGGGTTCAC**					
Cysbar4 (2)	KT344786	F:HEX-TAGTTGACTGTTTTGCCGCC	(GTT)_10_	75–149	23	0.439	0.216***
		R:ACCGTACAAGAGTGTTGCTG					
**Cysbar5 (1)**	**KT344787**	**F:HEX-AATCAGAAGACGGCGAATGC**	**(CA)**_**12**_	**134–205**	**39**	**0.736**	0.255***
		**R:GCGTACCGACTCTTTAGGTG**					
**Cysbar6 (2)**	**KT344788**	**F:6FAM-ACGAAGCCTCTCTATTTTGCC**	**(CAG)**_**7**_	**83–102**	**9**	**0.585**	− 0.710***
		**R:CCGACTAAAAGCATCGTCCC**					
Cysbar7 (2)	KT344789	F:ROX-AGGGGTGCTACAGATGATGC	(CA)_14_	132–165	16	0.538	0.069
		R:TCTCAGAAATCCAGCCCGTG					
Cysbar8 (2)	KT344790	F:6FAM-CGTATGGTAGGCATGCTTGG	(TG)_13_	142–172	16	0.593	**− **0.015
		R:CGTATAACACCTGCCTTTAGGAAC					
Cysbar9 (2)	KT344791	F:DRAGONFLY-CGACGTAGCATCGTGTTGTC	(TG)_15_	168–193	10	0.329	0.307**
		R:GCTAGAACGGTTTGGTGCTG					
Cysbar10 (1)	KT344792	F:6FAM-GTATGGCTCTACTCCCACCC	(AGT)_10_	234–254	10	0.549	0.123***
		R:GCATTTTTGCATTCTACCGGC					
Cysbar11 (1)	KT344793	F: DRAGONFLY-AGGCACCCTGGATACTGTTG	(GTA)_7_	240–257	5	0.449	0.220***
		R: ATCGCAGTTGATTTCCGCAG					
Cysbar12 (2)	KT344794	F:6FAM-TGCTTTACTGAAACCCCGTC	(CCA)_8_	215–243	11	0.326	**− **0.003
		R:TCGATCTATGTCGTCAGGGC					

N_a_: Number of alleles, H_S_: Nei’s genetic diversity within subsample; F_IS_: fixation index and its *p*-value from U test for heterozygote deficiency or excess (**p-value < 0.01, ***p-value < 0.001, non-significant for all the other loci). The three loci bolded were removed from the final dataset.

The analysis of 375 *G. barbata* individuals genotyped with twelve microsatellite markers showed significant linkage disequilibrium between two loci, cysbar6 and cysbar12, after Benjamini and Yekutieli adjustment. We removed three problematic microsatellite markers, Cysbar3 and Cysbar5 for which technical issues were observed, and Cysbar6, in linkage disequilibrium with Cysbar12, from the final dataset.

**Figure 2:**
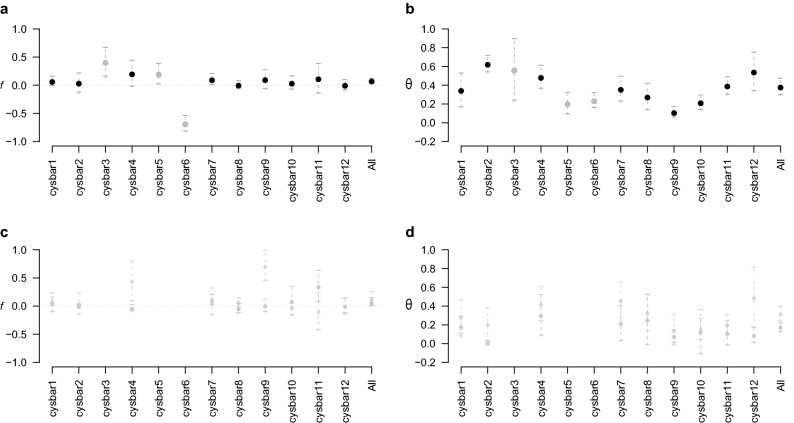
Variation of Weir and Cockerham^120^ estimation of F_IS_ (*f*) and F_ST_ (*θ*) for each of the twelve microsatellite markers and across loci in *Gongolaria barbata* sampled in the Ionian, Adriatic and Black Seas (**a** and **b**, respectively). The variation of Weir and Cockerham^120^ estimation of F_IS_ (*f*) and F_ST_ (*θ*) for the nine microsatellites used in the study within the Ionian and Adriatic Seas (in pink) and within the Black Sea (in blue) was also provided (**c** and **d**, respectively). For each locus, 95% confidence intervals were obtained by jackknife over subsamples and by bootstrap over loci. The averaged *f* and *θ* across loci were computed based on nine microsatellite markers, i.e. removing the three markers shaded in grey. The horizontal dashed line represents the 0 value. This figure was created using the R software^107^.

Once removing these three markers (N = 375 individuals, nine microsatellite markers, 6.7% missing data, SI1), we analyzed the stability of F_IS_ and F_ST_ from one locus to the other following the determination key proposed by De Meeûs^68^. A strong and significant heterozygote deficiency was observed with *f* = 0.066 (*p*-value = 10^–3^). The F_IS_ variation across loci was moderate (Fig. 2), but a high F_ST_ variation was observed among loci, the Pearson correlation between F_IS_ and F_ST_ was not significant (r(F_IS_,F_ST) =_ 0.257 *p*-value = 0.397) and the standard error of F_IS_ (StrdErrFIS = 0.022) was two times lower than for F_ST_ (StrdErrFST = 0.046). A strong and significant correlation was however observed between the number of missing data and F_IS_ values (ρ = 0.733, *p*-value = 0.016). According to the determination key proposed by De Meeûs^68^, null alleles may have persisted in the dataset, despite a careful attention paid to minimize them (cf. analyses aforementioned), which would explain these results. This hypothesis cannot be ruled out since null homozygotes (i.e. non-amplifying locus of an individual, while all other loci successfully amplify in this individual) were still present in the final dataset. Heterozygote deficiencies may also be explained by demographic causes, such as a Wahlund effect.

### A similar level of genetic diversity among samples

Estimates of allelic richness, gene diversity and departure from Hardy–Weinberg equilibrium, for each study site are presented in Table 1. Allelic richness (A_r_) ranged from 1.9 (site 14) to 3.5 (site 3), and expected heterozygosity (H_S_) from 0.272 (site 14) to 0.611 (site 11). A_r_ and H_S_ did not differ significantly among subsamples (A_r_: *p*-value = 0.17; H_S_: *p*-value = 0.20).

Departure from Hardy–Weinberg equilibrium was found in six study sites (sites 3–5, 7 and 13).

Multi-Locus Heterozygosity (MLH) ranged from 0 (sites 6, 7, 12 and 13) to 8 (sites 1, and 7). MLHs were normally distributed (Shapiro test *p*-values > 0.5) and no difference in MLH distribution was observed (ANOVA *p*-value > 0.5). Identity disequilibrium, i.e. a measure of departure from random associations of homozygosity between loci, was however identified in site 4, with the only significant g_2_ observed (Table 1), suggesting that few inbred individuals were present in this subsample. Selfing rate was estimated within each subsample and did not differ significantly from zero (*p*-values > 0.1 after Benjamini and Yekutieli adjustment).

### Strong genetic structure at large spatial scale

High and significant genetic differentiation was observed among all subsamples across loci (θ = 0.375, *p*-value < 10^–3^, ENA corrected F_ST_ = 0.355, IC = [0.283, 0.440]). Based on a hierarchical analysis with three levels (level 1: Mediterranean and Black Seas, level 2: Ionian, Adriatic and Black Seas, level 3: all subsamples), we observed that the three levels influenced genetic structure at a 5% level (F_lev1/Total_ = 0.137, IC = [− 0.033, 0.322]); F_lev2/lev1_ = 0.279, IC = [0.196, 0.350]); F_lev3/lev2_ = 0.142, IC = [0.144, 0.250]); F_Ind/lev3_ = 0.066, IC = [0.027, 0.109]).

**Figure 3:**
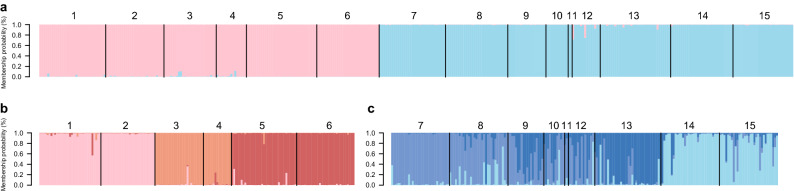
Genetic population structure of *Gongolaria barbata* based on nine microsatellite markers. Individual Bayesian ancestry proportions were determined using Structure with K = 2 across *G. barbata* distribution range (**a**), with K = 3 (**b**) within the Ionian and Adriatic Seas and with K = 3 (**c**) within the Black Sea. Black lines separate each study site. The clusters identified are distinguished by pink and blue colors, standing for the Mediterranean and Black Sea sites, respectively and referring to Fig. 1. Each individual is depicted as a vertical bar with colors distinguishing its ancestries to the clusters identified. Individuals’ assignment was visualized using the R software^106^.

The Structure analysis revealed a net differentiation between the Mediterranean and the Black Seas samples (Fig. 3a), in line with the strong and significant heterozygote deficiencies observed, partly explained by the presence of null allele, but also likely explained by a Wahlund effect, which justified a clustering approach. This result is congruent with the Principal Component Analysis along the first axis (PCA; SI2) and the genetic distances (Table 3). Note that different values of K were explored (from two to 18) and K = 2 provided the best meaningful result, not only using the Evanno et al*.*^69^ method (ΔK_K=2_ = 4558.9, ΔK_K>2_ < 1.5), but also by crosschecking these results with PCA and F_ST_ estimates. Further increases in K were provided in SI2.

**Table 3:**
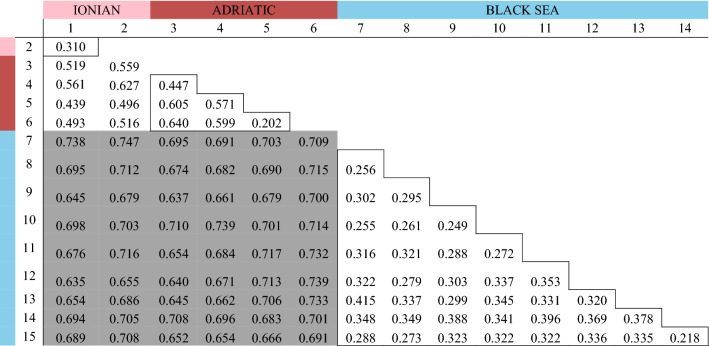
Pairwise genetic distances estimated using Cavalli-Sforza and Edwards^137^ for each pair of populations using the ENA correction^71^.

Genetic distances in squares underlined values among sites sampled within a Sea (e.g. within the Ionian, Adriatic or Black Sea). Mediterranean Seas *vs*. Black Sea genetic distance estimates were highlighted in grey.

These two lineages, i.e. Mediterranean samples and Black Sea samples, required being analyzed separately.

### Genetic structure within the Ionian and Adriatic Seas

A strong and significant heterozygote deficiency was observed with *f* = 0.103 (*p*-value < 10^–3^). High and significant genetic differentiation was observed among all subsamples across loci (θ = 0.313, *p*-value < 10^–3^, ENA corrected F_ST_ = 0.278, IC = [0.208, 0.341]). The F_IS_ variation across loci was higher than the variation observed among all subsamples (Fig. 2), the Pearson correlation between F_IS_ and F_ST_ was not significant (r(F_IS_,F_ST) =_ − 0.133 *p*-value = 0.733), and the standard error of F_IS_ (StrdErrFIS = 0.066) was 1.5 higher than for F_ST_ (StrdErrFST = 0.043). No correlation was observed between the number of missing data and F_IS_ values (ρ = − 0.017, *p*-value = 0.517).

Further, within the Ionian and Adriatic Seas, a strong structuration was revealed by the clustering analyses. Again, different values of K were explored (from two to ten, results provided in SI3) and K = 3 (Fig. 3b) provided the best meaningful results (ΔK_K=3_ = 1551.4, ΔK_K=4_ = 112.6, ΔK_K=2, 5–10_ < 1, but also by crosschecking these results with PCA and F_ST_ estimates). Geographic groups comprised (i) sites 1–2, (ii) sites 3–4 and (iii) sites 5–6. A similar genetic structure was detected by PCAs, provided in SI3.

### Genetic structure within the Black Sea

A moderate but significant heterozygote deficiency was observed with *f* = 0.052 (*p*-value = 10^–3^). High and significant genetic differentiation was observed among all subsamples across loci (θ = 0.169, *p*-value < 10^–3^, ENA corrected F_ST_ = 0.163, IC = [0.111, 0.207]). The F_IS_ variation across loci was moderate (Fig. 2), the Pearson correlation between F_IS_ and F_ST_ was not significant (r(F_IS_,F_ST) =_ 0.311, *p*-value = 0.300), and the standard error of F_IS_ (StrdErrFIS = 0.040) was c.a. two times higher than for F_ST_ (StrdErrFST = 0.026). No correlation was observed between the number of missing data and F_IS_ values (ρ = − 0.167, *p*-value = 0.666).

Genetic structure within the Black Sea was not depicted as clearly as in the Mediterranean Sea, revealing higher levels of gene flow among sampled sites. Based on the clustering analyses, at K = 3 (Fig. 3c; ΔK_K=3_ = 117. 44), (i) sites 14 and 15, (ii) sites 7–8 and 10, and (iii) site 13 showed clear differentiation among them, though some individuals were genetically assigned to the alternative genetic backgrounds (Fig. 3c). Further, sites 9–12 showed mixed ancestry to three genetic backgrounds, suggesting some local gene flow. In addition to mixed ancestries observed in sites 9–12, some individuals were assigned to one of the two genetic backgrounds with an ancestry rate higher than 0.8. Further increases in K did not result in new meaningful clusters (ΔK_K_ < 11, except ΔK_K=2_ = 101.3, results provided in SI4). Clustering from PCA (provided in SI4) appeared similar to clustering revealed by Structure.

### Within sea estimates of dispersal

**Figure 4:**
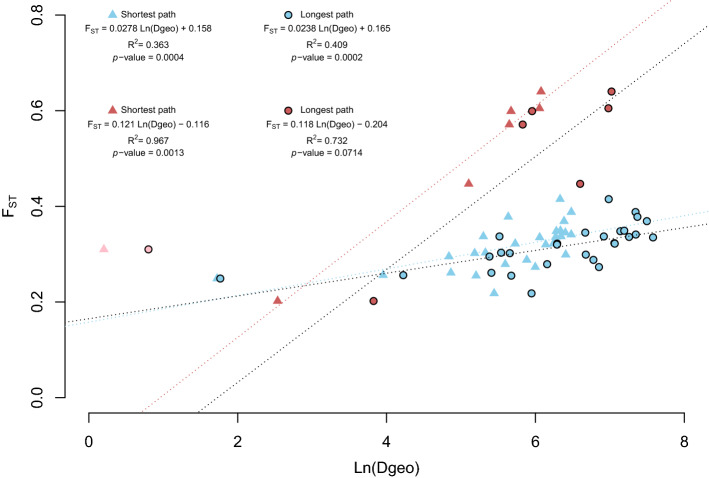
Genetic Isolation by distance in *Gongolaria barbata* in the Adriatic and Black Seas based on the shortest (diamond) and the longest path (circle). Distances displayed are site pairwise F_ST_ (y-axis) and natural logarithm of oceanographic distances (x-axis). IBD was performed without the sampling site 11 in the Black Sea. This figure was created using the R software^107^.

Significant correlations were observed between genetic differentiation and oceanographic distances among sites, either using the shortest path, i.e. the shortest distance between two sites, or the longest path, i.e. following the coastlines, in the Black Sea, but only using the shortest path in the Adriatic Sea (Fig. 4). In the Adriatic, we observed a slope b = 0.121, which, once translated, suggested a relatively small neighborhood size N_b_ = 8 individuals and an immigration from neighbors within the same sea of N_e_m = 1.32 individual per generation and subpopulation. With an averaged N_e_ = 78.55, and based on an estimated number of populations between 10 and 100 in the Adriatic Sea, the estimates of effective population density ranged from 0.006 to 0.06 individuals/km^2^, i.e. very sparse populations, and the dispersal (δ) was estimated between 186 and 587 m, i.e. very restricted dispersal. In the Black Sea, based on the longest path that better explained the regressions, the slope b = 0.0238 was translated into a neighborhood size N_b_ = 42 individuals and an immigration from neighbors within the same sea of N_e_m = 6.69 individuals per generation and subpopulation. With an averaged N_e_ = 50.76, and based on an estimated number of populations between 100 and 1000 in the Black Sea, the estimates of effective population density ranged between 0.014 and 0.140 individuals/km^2^, i.e. very sparse populations, though higher than in the Adriatic, and the dispersal (δ) was estimated between 129 and 408 m, i.e. very restricted dispersal as estimated in the Adriatic.

## Discussion

In the present study, we investigated the genetic diversity and structure of fifteen remnant populations of *G. barbata* sampled in three seas, the Ionian, the Adriatic and the Black Sea, aiming to evaluate whether the natural recovery of disturbed populations may occur without the help of restoration actions.

The use of microsatellites for conservation genetic purposes has undoubtedly be a major advance, providing several advantages over allozymes as nuclear markers^65,70^. But a variety of potential pitfalls need to be carefully considered when using microsatellites, especially regarding null alleles^67,68,71,72^. The determination key^68^ applied on the microsatellite markers specifically developed for *G. barbata* allowed us interpreting data with heterozygote deficits and discriminating technical to demographic causes. Facing *G. barbata* population decline, low level of intra-population genetic variation would be expected due to recent demographic bottlenecks in wild populations. Interestingly, the polymorphism observed in *G. barbata* is on the same range than that observed not only in other *Cystoseira* species^54,57,61^, but also, in other Fucales^73–75^. Across the fifteen *G. barbata* sampled sites, we found significant heterozygote deficiency, which is common in *Cystoseira*^54,56,58^. However, in most previous published studies, markers with potential technical issues were not removed nor discussed, excluding the possibility of direct comparisons among species regarding the possible causes explaining these deviations.

Here, the technical issues of the microsatellite markers were ruled out and allele frequencies corrected to consider the presence of null alleles, leaving the origin of the significant deviations from HW expectations to be biological, such as inbreeding or related to a Wahlund effect. Most Fucales are monoecious, bearing hermaphroditic conceptacles so that self-fecundation may occur^63,76–78^. Because all *Cystoseira* s.l. are considered to be self-compatible hermaphrodites with extremely restricted gamete dispersal, inbreeding may likely arise from the mating among relatives or selfing. In *T. elegans*, based on allele frequencies corrected considering the presence of null alleles, departures from HWE equilibrium in all three Catalan populations were interpreted as resulting from inbreeding^79^*.* Likewise, in *E. amentacea,* based on allele frequencies corrected considering the presence of null alleles, departures from HWE are likely explained by selfing or between neighboring individuals that are expected to be more related on average^55^. In the present study, however, departures from HWE in *G. barbata* cannot be explained solely by the presence of inbred individuals: inbreeding was detected in a single site located in the Adriatic Sea. In addition, we did not detect selfing from the analyses of the multilocus heterozygosity distributions. In *Cystoseira s.l.*, fertilization is oogamous, with thousands of biflagellate male gametes meeting female gametes in the vicinity of hermaphroditic conceptacles grouped within terminal receptacles. This reproduction mode strongly suggests that selfing may occur in these species if gametes are self-compatible. But, we could not find solid evidence of self-compatibility in the literature, or direct citation of an experimental study specifically testing for it^55,56,80^, a common assumption that should therefore deserve further investigation. Given this, mating among relatives would be more likely, without solid evidence of self-compatibility in *Cystoseira *s.l., facilitated by the very low dispersal of the fertilized eggs^29^, leading to clustering of related individuals^55^. This said, deviations from HW expectations may also be attributed to a Wahlund effect: given the very limited dispersal abilities of the species^29,30^, mixing of populations that differ in allele frequencies would result in significant heterozygote deficiencies in the pooled population, a scenario that is very likely, especially if more related individuals settle close to each other so that populations are made of a sum of different families.

One of the most expected results was certainly the strong genetic structure observed across the three seas, with the convergence of all methods towards nearly no gene flow between the Black Sea and the Ionian and Adriatic Seas. Strongly differentiated populations have been observed not only in other *Cystoseira *s.l. species overall their distribution ranges^54^, but also in other canopy-forming seaweeds^81–83^. The genetic distinction of the Black Sea populations is very likely explained by a lack of exchange of migrants through the Bosphorus strait, consistent with previous finding based on mtDNA markers^62^. A very similar structuration pattern was found over the same sampled sites in *Tritia neritea*, a gastropod characterized by a direct cycle without a pelagic larval stage^84^. The restricted exchange of water masses and the difference in water density salinity and temperature between the water masses of the Mediterranean and the Black Seas across the Bosphorus strait has already been reported as a main driver of genetic structure in various species^84–89^. Besides the environmental gradients, transition zones are often zones of secondary contact of two lineages, i.e. a zone of contact between lineages previously isolated into glacial refugia^90^. Current data make it difficult to establish the historical demographic contexts of the observed genetic divergence, the identification of any barriers to gene flow that promote/maintain genetic isolation, as well as the exact spatial segregation of the two main genetic clusters in *G. barbata*. Given the observed differentiation between the Mediterranean and Black Seas populations, both using nuclear and mitochondrial markers^62^, and the inter-basins context, this pattern is likely resulting from divergence without gene flow. The extent to which these two genetic backgrounds are reproductively isolated deserves further attention which could be addressed, for instance, with a study covering the entire range distribution of the species, and especially covering the contact zones to identify barrier to gene flow^91^. The presence of reproductively isolated lineages would prevent the success of genetic rescue if the populations crossed were found too divergent to reproduce. Genome-wide data will likely help to delineate the presence of reproductively isolated lineages and test the hypothesis of one or several evolutionary species^92^. Similar and high genetic diversity was nevertheless observed in these two genetic backgrounds, supporting long-term regional persistence of large populations rather than recent expansions and isolation by distance, which are often associated with loss of diversity in fucoids^93^.

In addition to the strong differentiation between the Mediterranean and Black Seas observed in *G. barbata*, genetic analyses revealed high genetic structure at fine spatial scale, indicative of limited population connectivity, even between sampled sites separated by few kms. Such levels of genetic differentiation are in the range of that observed among populations of *Cystoseira *s.l. studied over the similar spatial scales (i.e. within seas) using microsatellite markers^11,55,56,79^, suggesting similar dispersal abilities of *G. barbata* to other *Cystoseira *s.l. The estimated dispersal distance per generation in *G. barbata* was extremely small, likely ranging from 129 to 587 m across the Adriatic and the Black Sea. Although these estimated dispersal distances should be carefully interpreted^94,95^, these estimations were nonetheless comparable to distances estimated from spatial autocorrelation analyses in *E. amentacea*^55^, and in agreement with estimated distances from field observations^29^. These estimated dispersal distance were likely smaller than the distance between two consecutive suitable habitat patches (shallow sheltered coasts and lagoons), enabling the migration to reverse the negative effect of genetic drift. Our results suggest that, so far, *G. barbata* isolated populations did not seem to suffer from inbreeding depression, and displayed intra-population genetic variation similar to other Fucales; genetic and demographic monitoring should be pursued to address the most appropriate conservation issues, e.g. assisted gene flow, minimizing non-genetic threats, or in case of small inbred populations, genetic rescue with highly divergent populations^50^.

Pairwise genetic differentiation among sampled sites were significantly correlated with geographic distances in the Black Sea and Adriatic. Such isolation-by-distance (IBD) is common in macroalgae, whatever their algal division, habitat and life cycle^96^, and usually observed in Fucales^97–99^. However, in *E. amentacea*, genetic structure was not significantly related to geographic distances; this relationship was improved when dispersal probability across available rocky habitat was estimated by simulating stepping-stone directional transport mediated by ocean currents^55,58^. Considering the very low dispersal life history of *E. amentacea*, dispersal in this species would be facilitated by possible transport in floating rafts. Indeed, rafting is a relatively frequent and documented mechanism of marine dispersal allowing fragments of thalli to be transported entangled in floating rafts of other algae^100^. In *G. barbata*, relative long dispersal could also occur through drifting of fertile individuals or ramification, bearing fertile receptacles, thanks to their aerocysts, helping in preserving inter-population genetic variation. Greater connectivity was, for instance, observed in *C. compressa* compared to *E. selaginoides* and *E. amentacea*, which was ascribed to the presence of aerocysts, providing a buoyancy property that *E. selaginoides* and *E. amantacea* do not possess^54^. Dispersal as fertilized zygotes has also been observed in *Cystoseira *s.l. under strong hydrodynamic events^58,100^ but would not allow such long-distance dispersal. Such properties would enhance connectivity among suitable habitat patches in *G. barbata*, especially within the Black Sea as compared to the Adriatic Sea. Indeed, while differences in the range of geographic distances among sampled populations may partially explain this result, this finding could also reflect: (1) the presence of more free-living individuals within the Black Sea as compared to the Adriatic, (2) the higher homogeneity of water masses in the Black Sea; the oceanographic circulation is composed of a counter-rotating (cyclonic) Rim Current surrounded by many predominantly anticyclonic coastal eddies^101^ while the three cyclonic gyres (the north, the central and the southern Adriatic sub-gyres) and the physical and biogeochemical features operating in the Adriatic Sea creates natural barriers for dispersion^102^ and (3) larger effective population size in the Black Sea, in line with higher abundances in the Black Sea than in the Mediterranean Sea, these three hypothesis being non-exclusive. Nevertheless, despite potential occasional long-distance gene flow through rafting or drifting, strong genetic differentiation among populations together with homozygotes deficiencies suggest very restricted connectivity among natural populations of *G. barbata*, a result coherent among *Cystoseira *s.l. studies^54–56,58^. *G. barbata* populations are likely connected via neighboring subpopulations in the form of stepping stones dispersal, as supported by significant IBD. Considering the limited dispersal estimates found here, our results call into question the natural recovery of disturbed populations if suitable patches of habitat are more than 1 km far apart.

The restricted connectivity observed among *G. barbata* subpopulations suggests that each population represents a different evolutionary significant unit, giving the highest conservation priority to this threatened species^103^. Conservation should be designed to preserve the distinctive genetic diversity here observed. However, conservation efforts must be complemented by a reflection considering all the management units and their exchanges through gene flow, e.g. genetic rescue by assisted gene flow, to counteract genetic diversity erosion within each evolutionary significant unit. Other active strategies should be considered to restore the connectivity between these currently isolated populations, fostering the natural recovery of degraded populations (e.g. limiting the regression causes) or actively restoring lost populations^15,17,19,20^. Regarding the very restricted dispersal abilities, more genetic data on *Cystoseria* based on a sampling not only covering the large but also the very fine-scale distribution ranges and across time are needed to conserve the iconic Mediterranean and Black Seas marine forests and upscale reasoned ecological restoration programs.

## Methods

### Sampling collection and DNA extraction

*Cystoseira *sensu lato corresponds to three monophyletic clades recognized as three different genera^62,104^. The two clades outside the *Cystoseira *sensu stricto clade were recently better redefined, so that the formerly-known *Cystoseira barbata* was first renamed *Treptancha barbata*^104^ then *Gongolaria barbata*^62^*. Gongolaria barbata* samples were collected by snorkeling in 2013 in 15 sites along the coasts of the Ionian, Adriatic and Black Seas, (Fig. 1; Table 1). Individuals were collected at least 50 cm away from each other, and only the apical part of a primary ramification (ten centimeters) was collected and preserved in dried silica gel for future DNA analysis. The map (Fig. 1) was created using the R package mapdata^105–107^.

Genomic DNA was extracted from 10 mg of lyophilized tissue using DNeasy 96 Plant Kit (Qiagen) following manufacturer’s instructions.

### Microsatellite development

Genomic DNA of 15 individuals from different locations in the Mediterranean and Black Seas was sent to the Ecogenics GmBH company (http://www.ecogenics.ch), commissioned to identify and develop a set of twelve Single Sequence Repeats (SSRs), i.e. microsatellite markers. Briefly, size selected fragments from genomic DNA were enriched for SSR content by using magnetic streptavidin beads and biotin-labeled CT and GT repeat oligonucleotides. The SSR-enriched library was analyzed on a Roche 454 platform using the GS FLX Titanium reagents. A total of 24,770 reads were sequenced with an average length of 474 base pairs. Of these, 3008 reads (12.2%) contained a microsatellite insert with a tetra- or a trinucleotide of at least six repeat units, or a dinucleotide of at least ten repeat units. Suitable primer design was possible in 1997 reads. Twelve markers (0.6%) showed a correct amplification and variability by polymerase chain reactions (PCR).

Following PCR optimization and polymorphism analysis of each of these twelve primer pairs, a multiplex procedure was performed using Multiplex Manager^108^ and four combinations of multiplex were tested. Based on banding patterns checked on agarose and polyacrylamide sequencing gels (2% and 8%, respectively), two multiplex reactions of six microsatellites with forward primers labeled with ABI fluorescent dyes (Table 2) showed clear banding patterns and were selected for routine genotyping on a capillary sequencer.

### Microsatellite genotyping

Individuals were genotyped using these twelve microsatellite markers newly developed as follow: PCR amplifications were done in a final volume of 25 µl containing 5–15 ng of genomic DNA, 1X Master Mix (Type-it kit, Qiagen) and 0.1 μM of each primer. DNA amplification started with a 5 min denaturation at 95 °C, followed by 30 cycles comprising a denaturation of 30 s at 95 °C, a hybridization of 90 s at 60 °C and an elongation of 30 s at 72 °C and finished with a last elongation step at 60 °C for 30 min. PCR products were genotyped using an ABI 3730XL capillary sequencer at Macrogen Europe Inc. (Amsterdam, the Netherlands). Alleles were scored using GeneMapper v. 4.0 (Applied Biosystems) using an internal size standard (Genescan LIZ‐500, Applied Biosystems) and manually checked. Genotypes of every individual were checked twice with identical results.

### Testing the dataset quality

Before further analysis, we checked for clonal propagation across all samples by estimating the probability of identity (PI, i.e. the probability that two independently sampled individuals within a mating population share an identical multilocus genotype by chance) using GenAlEx v6.502^109^. With a PI < 1.8 10^–5^ per population, any identical genotypes found in the dataset were presumed to be clones. These clones could either belong to a unique genet or be two representatives of the same clone that have spread. To discriminate between these alternative hypotheses, we carefully checked the sampling site(s) of the two individuals presumed as clones and their label numbering, used as a proxy of the distance between these individuals.

For each pair of loci, linkage disequilibrium (LD) was tested using the G-based test implemented in Fstat 2.9.4^110^, which was proven to be the most powerful approach to combine tests over all subsamples^111^. The 66 tests conducted (12loci × (12–1)/2) were not independent and *p*-values were corrected using the Benjamini and Yekuteli procedure^112^ to obtain FDR (false discovery rate) corrected individual *p*-values.

MicroChecker software^113^ was used to detect the presence of null alleles and eventual scoring errors (due to stuttering and/or large allele dropout/short allele dominance). We reported the null allele frequencies estimated using the Bookfield's second method provided by MicroChecker, and computed (1) null alleles frequencies expected under Hardy–Weinberg Equilibrium, and (2) the number of null alleles expected based on these frequencies and the number of individuals genotyped. We then tested the adjustment between observed and expected numbers of missing data^67,71^ with a unilateral exact binomial (H1: there is less missing data observed than expected). Additionally, we tested for genotyping errors due to the occurrence of short allele dominance (SAD) using unilateral (ρ < 0) Spearman's rank correlation tests between allele size and F_IS._ These tests were also performed with F_IT_, aiming to increase the power of statistical tests^71^. Genotyping errors due to the occurrence of stuttering were tested following De Meeûs et al*.*^67^.

### Analyses of genetic diversity

Allele frequencies, the average number of alleles (N_all_), allelic richness (A_r_), gene diversity (H_S_) and the fixation index (F_IS_) were estimated for each study site using Fstat 2.9.4^110^. Briefly, A_r_ is the expected number of alleles in a random subsample of size *g* drawn from the population^114,115^. This rarefaction method allows evaluation of the expected number of different alleles among equal sized samples drawn from several different populations. Genetic diversities between two groups were compared using bilateral tests with 10,000 randomizations implemented in Fstat 2.9.4^110^. We tested for heterozygote deficiency across loci and subsamples using the U test^112^ implemented in Genepop 4.7.5^116^.

To test for possible inbreeding occurring within each sample, individual Multi-Locus Heterozygosity (MLH) was calculated for each individual. In a population with variance in inbreeding, inbred individuals are less heterozygous (i.e. lower MLH) than non-inbred ones. Inbreeding variance generates Identity Disequilibria (ID)—i.e. correlations in homozygosity across loci, a measure of departure from random associations between loci^117^. IDs were measured for each sampling site by calculating the g_2_ parameter and its standard deviation using the RMES software^118^. The g_2_ parameter measures the excess of double heterozygotes at two loci relative to the expectation under a random association, standardized by average heterozygosity, providing a measure of genetic association and inbreeding variance in the population, free of technical biases. To test for the significance of g_2_, random re-assortments of single-locus heterozygosities among individuals were tested using 1000 iterations. Selfing rate within each subsample was also estimated using RMES^118^. To test whether the selfing rate significantly differed from 0, both the unconstrained and constrained likelihood options were run. We then computed Δdev = 2 * (lnl(unconstrained) − lnl(constrained)) and compared it to a chi-square with one degree of freedom^118^.

### Analyses of genetic structure

First, we estimated population genetic structure through the approach developed in De Meeûs^68^ based on the Wright's fixation indices^119^, F_IS_, which measures inbreeding of individuals relative to inbreeding of subsamples, F_ST_, which measures inbreeding of subsamples relative to total inbreeding, and F_IT_, which measures inbreeding of individuals relative to total inbreeding. In other words, F_IS_ measures the deviations form Hardy–Weinberg equilibrium (i.e. local panmixia), F_ST_ measures genetic differentiation between subsamples, and F_IT_ reflects the combination of both. Briefly, this approach is based on the examination of the F-statistics stability across loci and subsamples to interpret data with heterozygosity deficiencies. Heterozygosity deficiencies can arise from null alleles, stuttering, short allele dominance, allele dropouts (i.e. technical problems), Wahlund effect (the juxtaposition of several groups with different allele frequencies), and/or selfing and sib-mating. By examining the (1) missing data in the dataset (across loci and subsamples), (2) variations of F_IS_ and F_ST_ across loci, (3) standard error of jackknife over loci for F_IS_ (StrdErrFIS) and F_ST_ (StrdErrFST), and (4) the correlation between F_IS_ and F_ST_, De Meeûs^68^ proposed a determination key to help discriminating demographic from technical causes.

Wright's F-statistics were estimated through Weir and Cockerham's unbiased estimators^120^, i.e.* f*, *θ* and *F* for F_IS,_ F_ST_ and F_IT_ estimators, respectively. We computed the 95% confidence intervals of F-statistics using the StrdErrFIS and StrdErrFST computed by jackknife over subsamples, for each locus, and we used the 95% confidence intervals computed by bootstrap over loci. Correlations between F_IS_ and F_ST_ were measured using Pearson’s correlation coefficients in R^107^. All these analyses (parameters estimates, jackknife, bootstrap) were performed using Fstat 2.9.4^110^ and R^107^.

We further tested a correlation between F_IS_ and the number of missing data^71^: the significance of correlations was tested with a unilateral (ρ > 0) Spearman's rank correlation test in R^107^.

In addition to the aforementioned approach, genetic structure among sampling sites was depicted using more classical tools, aiming to look whether geographic differentiation exists. We used three different approaches: (1) F_ST_ estimates (2) Principal Component Analyses (PCA) computed on the matrix of genotypes, and (3) an individual-based Bayesian clustering method. Comparing results from analyses using different statistical approaches allows us to make solid assumptions about our data: it allowed us to cross check the outputs of a model-based approach with strong priors and hypotheses (HW equilibrium, no linkage between markers) as implemented in Structure 2.3.4^121,122^, with methods based either on distance with few (nearly no) assumptions on data (i.e. PCA) or on distance matrices among sets of individuals that fall within predefined population samples. Estimates of F_ST_ were calculated using FreeNA with the ENA correction^72^. To test for genetic isolation among subsamples, we used the G-based test over all loci. The PCAs were carried out using the R package adegenet 1.4–2^123^. The individual-based Bayesian clustering analysis was performed with the software Structure 2.3.4^121,122^. For each value of K (ranging from 1 to 18), 30 replicate chains of 200,000 Markov Chain Monte Carlo iterations were run after discarding 25,000 burn-in iterations. An admixture model with correlated allele frequencies was applied with a priori information on sample origin. For each value of K, 30 output files were produced by Structure 2.3.4 with slightly different ancestry proportions; using Clumpp^124^, we determined individual ancestry proportions (*q*-values; i.e. assignment probabilities) that best matched across all replicate runs for each K value. We visualized individuals’ assignment in the R software^107^.

We estimated and tested hierarchical F-statistics using the HierFstat package^125^ with three levels, the Mediterranean *vs.* the Black Seas subsamples (level 1), the Ionian, Adriatic and Black Seas (level 2), and all subsamples (level 3).

### Estimates of larval dispersal

Finally, we tested for the presence of genetic Isolation-By-Distance (IBD) by investigating the relationship between using Cavalli-Sforza and Edward’s chord distance^126^ (Table 3) and the natural logarithm of oceanographic distances (Ln(Dgeo)^127^. Pairwise oceanographic distance between sampled sites was estimated within each sea as the shortest oceanographic distance according to the ‘Vincenty (ellipsoid)’ method using the geosphere package^128^. However, as the shortest path does not reflect the sea hydrodynamics^129,130^, pairwise oceanographic distances between sampled sites were also estimated following the coastlines, calculated by summing all the straight lines connecting each cape on a map at 1:500,000 on QGis 3.18. For the Black Sea, which is circular, having the choice between following the movement against the clock or clockwise to calculate the coastline distance between two populations, only the shortest distance was kept.

Regressions between genetic and geographic distances as described above were investigated within the Adriatic on the one hand and within the Black Sea (excluding subsample 11) on the other hand, using Fstat^110^. With significant IBDs, the IBD slope allows estimating the neighborhood N_b_ = 1/b and the number of immigrants from neighbors N_e_m = 1/(2πb) = 4πD_e_σ^2^, with D_e_ the effective population density and σ^2^ the average of squared axial distances between adults and their parents^131,132^. To estimate D_e_ and σ^2^, we first estimated the effective population size N_e_ within each Sea (Adriatic and Black Seas): we measured the non-random association of alleles at different loci within F1 population^133^ using a bias correction^134,135^ implemented in NeEstimator v2 software^136^. The P_crit_ was adjusted following recommendations^135^ and random mating was applied. Estimating N_e_ allows computing effective population densities D_e_, as the averaged N_e_ multiplied by the number of subsamples within a sea divided by the surface of the sea. The number of populations in the Adriatic Sea was estimated as follow: (1) *Gongolaria barbata* stands for 10% of the *Cystoseira *s.l. observed in the Mediterranean Sea^32^, (2) in the Adriatic, ca. 100 *Cystoseira *s.l. populations were recorded, (3) we estimated that 10% would be *Gongolaria barbata*, so that the number of populations in the Adriatic would be close to 10. Despite this estimation, we thought very low, we decided to test different number of populations, ranging from 10 to 100. In the Black Sea, the number of populations was estimated to be 100^63^, but we also tested different number of populations, ranging from 10 to 100. Once D_e_ computed, a rough proxy of dispersal (δ), in order of magnitude, can be computed as δ ≈ 2 (1/(4πDe × b))^−1/2^ (i.e. N_e_m = 4πDeσ2 <  =  > δ ≈ 2 (1/(4πDe × b))^-1/2^^126^).

## Supplementary Information

Supplementary Information.

## Data Availability

Microsatellite data has been deposited at DRYAD: 10.5061/dryad.g4f4qrfqs.
