# Supplementary material for: Highly restricted dispersal in habitat-forming seaweed may impede natural recovery of disturbed populations
Source: Sci Rep. 2021 Aug 18;11:16792. doi: 10.1038/s41598-021-96027-x (PMC8373921; doi:10.1038/s41598-021-96027-x)
Supplement: Supplementary file 1 — Supplementary Information. [file 41598_2021_96027_MOESM1_ESM.docx]

**Supporting Information of “Natural recovery of disturbed habitat-forming seaweed populations impeded by highly restricted dispersal”**

Florentine Riquet, Cécile Fauvelot, Christiane-Arnilda De Kuyper, Laura Airoldi, Serge Planes, Simonetta Fraschetti, Vesna Mačić, Nataliya Milchakova, Luisa Mangialajo, Lorraine Bottin

**Fig. SI1. Missing data per subsample and loci.** The percentage of missing data is provided per subsample (row) and per locus (column). The last column, "mean", represents the average percent of the population with missing data per locus. Identical numbers than in Fig. 1 and Table 1 are used for each study site.

**Fig. SI2. Genetic population structure** of *Gongolaria barbata* based on nine microsatellite markers. Principal Component Analyses depicted axes 1 and 2 (a), and axes 1 and 3 (b) with each label showing the barycenter of each study site. Identical numbers than in Fig. 1 and Table 1 are used for each study site. Individual Bayesian ancestry proportions were determined using STRUCTURE with K = 3 (c) to K = 5 (e). Black lines separate each study site. The clusters identified are distinguished by different colors, from pink to blue colors, referring to the Mediterranean and Black Sea sites, respectively. Each individual is represented by a vertical bar partitioned into colored sub-bars whose lengths are proportional to its estimated probability of membership for the K clusters (c-e).

Note that the axes of the PCAs explained a relatively low share of the total variance (7.75%, 4.28% and 3.32% % for the first three axes, respectively). By examining the allelic frequencies of the nine loci, the subsamples shared a very high proportion of polymorphism, with minor differences between individuals making the major part of the variance, as exemplified in cysbar1 of which allelic frequencies were provided hereafter (Fig. SI2-2). All the small differences from the shared polymorphism were mainly shared between neighboring subsamples, likely explaining i) the low share of variance along the axes along which these subsamples can be distinguished and ii) the clear separation between geographically separated subsamples on the PCA plots. This is congruent with the large proportion of shared nucleotides based on mtDNA (ref 53).

Fig. SI2-2 Allelic frequencies for the locus cysbar1 per subsample. Identical numbers than in Fig. 1 and Table 1 are used for each study site.

**Fig. SI3. Genetic population structure** of *Gongolaria barbata* in the Mediterranean Sea (Ionian and Adriatic) based on nine microsatellite markers. Principal Component Analyses depicted axes 1 and 2 (a), and axes 1 and 3 (b) with each label showing the barycenter of each study site. Identical numbers than in Fig. 1 and Table 1 are used for each study site. Individual Bayesian ancestry proportions were determined using STRUCTURE with K = 2 (c) and K = 4 (d). Black lines separate each study site and the clusters identified are distinguished by different colors. Each individual is represented by a vertical bar partitioned into colored sub-bars whose lengths are proportional to its estimated probability of membership for the K clusters (c-d).

**Fig. SI4. Genetic population structure** of *Gongolaria barbata* in the Black Sea based on nine microsatellite markers. Principal Component Analyses depicted axes 1 and 2 (a), and axes 1 and 3 (b) with each label showing the barycenter of each study site. Identical numbers than in Fig. 1 and Table 1 are used for each study site. Individual Bayesian ancestry proportions was determined using STRUCTURE with K = 2 (c). Black lines separate each study site and the two clusters identified are distinguished by different color. Each individual is represented by a vertical bar partitioned into colored sub-bars whose lengths are proportional to its estimated probability of membership for the two clusters.
